# Intersectionality and Its Impact on Microaggression in Female Physicians in Academic Medicine: A Cross-Sectional Study

**DOI:** 10.1089/whr.2022.0101

**Published:** 2023-06-26

**Authors:** Alyson K. Myers, Myia S. Williams, Renee Pekmezaris

**Affiliations:** ^1^ Division of Endocrinology, Department of Medicine, North Shore University Hospital, Manhasset, New York, USA.; ^2^ Donald and Barbara Zucker School of Medicine at Hofstra/Northwell, Hempstead, New York, USA.; ^3^ Institute of Health Systems Science, Northwell Health, Manhasset, New York, USA.

**Keywords:** microaggressions, intersectionality, academic medicine, imposter phenomenon, patient safety

## Abstract

**Introduction:**

The burden of microaggressions in the workplace is an ongoing stressor for female physicians in academic medicine. For female physicians of Color or of the Lesbian, Gay, Bisexual, Transgender, Queer, Intersex, Asexual community, this burden is even heavier due to the concept of intersectionality. The goal of this study is to assess frequency of microaggressions experienced by participants. In addition, to explore the associations between microaggression and individual outcomes, patient care practices and attitudes, and perception of pay/promotion equity.

**Methods:**

This was a cross-sectional analysis of female residents, fellows and attendings conducted from December 2020–January 2021 at Northwell Health across all specialties. One hundred seventeen participants replied to the study in REDCap. They completed questionnaires related to the topics of imposter phenomenon, microaggressions, gender identity salience, patient safety, patient care, counterproductive work behavior and pay and promotion equity.

**Results:**

A majority of the respondents were white (49.6%) and 15+ years out of medical school (43.6%). Around 84.6% of female physicians endorsed experiencing microaggressions. There were positive associations between microaggressions and imposter phenomenon as well as microaggressions and counterproductive work behavior. There was a negative association between microaggressions and pay equity or promotion. The small sample size did not allow for us to examine differences by race.

**Discussion:**

Although the number of female physicians continues to rise due to an uptick in female medical school enrollees, female physicians still must deal with the burden of microaggressions in the workplace.

**Conclusions:**

As a result, academic medical institutions must seek to create more supportive workplace for female physicians.

## Introduction

Health care has been rooted in structural racial and gender inequality; historically women and underrepresented minorities (URM), have been especially limited in joining the physician workforce due to discriminatory policies for medical school entrance. This was further worsened by the 1910 Flexner report, in which 5 of 7 black medical schools were closed, resulting in the loss of potentially 20–30,000 black doctors.^1^ Additionally, the Flexner Report led to the closing of all but one of the six female-only medical schools.^2^

Despite the integration of all-male schools in the late 1800s, the closing of all-female medical schools drastically decreased the number of female doctors.^3^ Well over a century later, for the first time ever, women comprised majority of students enrolled in medical school in 2017. Despite this advance, a higher proportion of women were more likely than their male counterparts to remain at the instructor or assistant professor level, thereby resulting in a lack of parity in promotion and pay.^4^ For women of Color there is a “double tax,” as persons of Color, regardless of gender, and are less likely to be promoted than whites.^5^

Discriminatory practices in medicine not only occur at the structural or institutional level but can also be found at the individual level in the form of both internal (imposter phenomenon) or external oppressive forces (microaggressions).^6^ Microaggressions are everyday intentional or unintentional comments or behaviors that are perceived as discriminatory toward/by marginalized groups. They are further characterized as microassaults, microinsults, or microinvalidation.^7^ For example, in the health care setting microaggressions can appear as female physicians often being referred to as the nurse or “too young to be a doctor” and female physicians of Color are often questioned about their language skills, asked “where you from?” and referred to as “angry Black women.”^8^

Over time, microaggressions can lead to both physical harm and psychological conditions including but not limited to depression, anxiety, and substance use.^9^ Hence, while all women face microaggressions, women with traditionally marginalized intersecting identities, such as race and sexual orientation, have been known to experience more forms of microaggressions, which leads to worse psychological and organizational outcomes. In 1989, Kimberly Crenshaw coined the term “intersectionality” to highlight how multiple marginalized identities such as race, gender, and class for example *intersect* to compound inequities for the same individual. More specifically,^10^ intersectionality acknowledges that traditional feminist or antiracist theory was not inclusive of women of Color.^11^ This concept of intersectionality requires an even deeper dive beyond race and gender to assess factors such as socioeconomic status, and sexual orientation.^12^

In this cross-sectional study, we examined the frequency of microaggressions experienced by female physicians. Also, we explored the associations between microaggression and individual outcomes, patient care practices and attitudes, and perception of pay/promotion equity. We hypothesize that majority of female providers have experienced microaggression sometime during their career, with older physicians and physicians of Color experiencing more microaggressions than younger and white female physicians. We hypothesized that microaggressions will be positively related to individual outcomes (imposter phenomenon and gender identity salience) (H1); negatively related to patient care practices and attitudes (H2); positively related to counterproductive work behavior (CWB) (H3) and lastly, negatively related to perceptions of pay and promotions equity (H4). CWB are voluntary behaviors that are intentionally perpetrated by an employee with the sole purpose of harming the organization, other employees, and clients/customers.^13^

## Materials and Methods

### Participants and recruitment

In this cross-sectional study, we limited participation to resident, fellow, or attending physicians who self-identified as female. A total of 117 female physicians completed the survey, of which a majority were white (49.6%), attending physicians (71.8%), 45 years or younger (64%), and had completed medical school 15 years ago or less (56.4%) (Table 1). Similarly, most female physicians reported experiencing microaggressions (84.6%) and were in medicine or medicine subspecialties (29.9%).

**Table 1.** Participant

*Demographics (***n*** = 117)*		
	*Count*	*%*
Level of training		
Resident	23	19.7
Fellow	10	8.5
Attending	84	71.8
Age		
25–35 years	42	35.9
36–45 years	33	28.2
46–55 years	25	21.4
Over 55 years	17	14.5
Years from med school		
5 years or less	28	23.9
6–10 years	20	17.1
11–15 years	18	15.4
15+ years	51	43.6
Race		
Asian	22	18.8
Black or African American	26	22.2
Mixed (parents are from two different racial groups)	5	4.3
White	58	49.6
Other	6	5.1
Ethnicity		
Hispanic	10	8.5
Non-Hispanic	107	91.5
Microaggression		
No	18	15.4
Yes	99	84.6
Specialty		
Anesthesia	2	1.7
Dermatology	5	4.3
Family medicine	2	1.7
Internal medicine	17	14.5
Internal medicine subspecialty	18	15.4
Pediatrics	11	9.4
PM & R	1	0.9
Psychiatry	5	4.3
OB-GYN	12	10.3
Ophthalmology	10	8.5
Other	12	10.3
Radiology	2	1.7
Radiology oncology	7	6
Surgery	3	2.6
Surgery subspecialty	10	8.5

Ob/Gyn, obstetrics and gynecology; PM & R, physical medicine and rehabilitation.

Data were collected from December 2020 to January 2021. A link to an anonymous electronic survey (REDCap) was sent out *via* email to female physicians by all residency and fellowship program directors as well as managers of several list servs (*e.g.,* Department of Medicine, Medical School, Female Equity Committee, Black Girls Rock!! Chat to capture results from a variety of medical and surgical specialties) at our Health System. Additionally, participants received two follow-up e-mails during the data collection period to increase responses. Since this was a cross-sectional study, when asked for informed consent, participants were asked “have you previously taken this survey.” Those participants who reported “yes” were automatically excluded from signing informed consent and taking part in the study.

Participants were asked to provide demographic data, including age, level of training, years from medical school, race, ethnicity, experience with microaggressions (yes/no), and specialty. Participants who had experience with microaggressions were asked an open-ended question (if yes, please provide an example) of their experiences with microaggressions (Supplementary Appendix SA). Other measures on imposter phenomenon, microaggressions, gender identity salience, patient safety, patient care, counterproductive work behavior and pay and promotion equity are discussed below. The study was approved by the Institutional Review Board (IRB no. 20-1152).

### Measures

*Microaggressions* were measured using the 25-item Gendered Racial Microaggressions Scale.^14^ A sample item included “Someone accused me of being angry when speaking calm.” Response choices ranged from 0 (not at all stressful) to 5 (extremely stressful) for stressful items; frequency items ranged from 0 (never) to 5 (once a week or more); and experience items ranged from 1 = I did not experience this event to 5 = I experience this event 7 or more times.

*Imposter phenomenon* was measured using the 20-item Clance Imposter Phenomenon Scale.^15^ Sample items included “I have often succeeded on a test or task even though I was afraid I would not do well before I undertook the task.” Response choices ranged from 1 = not true at all to 5 = very true.

*Identity salience* was measured using a modified version of the six-item Centrality subscale of the revised Multidimensional Inventory of Black Identity (MIBI-Regard).^16,17^ Rooted in identity theory, which states that an individual has a number of identities (*e.g.,* race, gender, sexual orientation, religion *etc.*), which are hierarchically ordered, and as such behaviors and choices will correspond to the most salient identity,^18^ this scale was designed to assess how one salient identity (in this case race) can influence behavior and interpersonal interactions with others on three subscales (centrality, regard (public/private), and ideology).^19^ Where necessary, subscale items were modified to include *woman/women* instead of “Black/Black people.” Sample items include “Overall, being a woman has very little to do with how I feel about myself.” Response choices ranged from 1 = strongly agree to 7 = strongly disagree.

*Patient care practices* were measured using the five-item patient care practices of the self-reported suboptimal patient care practices and attitudes scale.^20^ A sample item included “I found myself discharging patients to make the service ‘manageable’ because the team was busy.” Similarly, *patient care attitudes* were measured using three-item subscales, with sample items, including, “I paid little attention to the social or personal impact of an illness on a patient.” Response choices ranged from 1 = never to 5 = several times weekly. Participants also had a not applicable option.

CWB were measured using the 10-item short version of the Counterproductive Work Behavior Checklist (CWB-C).^13^ Sample items include, “purposely wasted your employer's materials/supplies.” Participants rated items on a 5-point Likert scale ranging from 1 = *never* to 5 = *every day.*

*Perceived pay equity and promotion* was measured with three items.^21,22^ “My salary is fair given my qualifications for my position” (qualifications), “My salary is fair in relation to all other faculty in my business school” (internal equity), and “My salary is fair in relation to faculty with comparable qualifications at other institutions.” Participants rated items on a 5-point Likert scale from 1 = strongly disagree to 5 = strongly agree.

### Data analyses

Data were analyzed with control variables (*e.g.,* race, age, and training level). Analyses were performed in SPSS version 27. We conducted correlation analysis to examine the relationship among all variables (Table 2). To test our hypotheses linear regression analyses were utilized, where the control variables were entered in Step 1 and the predictor variable entered in Step 2.

**Table 2.** Descriptives and Correlations Among Major Variables

	*1*	*2*	*3*	*4*	*5*	*6*	*7*	*8*	*9*	*10*
1. Race (T1)	—									
2. Age	0.20^*^	—								
3. Training	−0.04	0.58^**^	—							
4. Microaggression	−0.07	−0.11	−0.15	—						
5. Imposter syndrome	0.04	−0.43^**^	−0.34^**^	0.25^**^	—					
6. Gender identity salience	0.06	−0.02	0.06	0.05	0.08	—				
7. Patient safety	−0.0.09	−0.17	−0.13	−0.13	0.07	−0.14	—			
8. Patient care	−0.05	0.24	−0.16	0.06	0.06	−0.17	0.47^**^	—		
9. CWB	−0.03	−0.28	−0.24	0.68^**^	0.024	−0.28	−1.00^**^	—	—	
10. Pay and promotion equity	−0.09	−0.30^**^	−0.15	−0.38^**^	0.00	0.02	0.14	0.07	0.14	—
*N*	117	117	117	117	117	117	95	94	24	117
*M*	—	—		41.61	59.78	5.08	2.41	1.99	13.08	2.70
SD	—	—	—	15.07	17.79	0.87	1.48	1.23	3.66	1.07
*Α*	—	—	—	0.93	0.95	0.63	0.83	0.70	0.81	0.92

^*^ *p* < 0.05. ^**^p < 0.01.

CWB, counterproductive work behavior; *M*, mean; SD, standard deviation.

## Results

Variable descriptions and correlations are shown in Table 2. Of note, imposter phenomenon was negatively associated with age and years of training, suggesting that younger female physicians are more likely to experience imposter phenomenon. In addition, level of training was negatively related to imposter phenomenon (*r* = −0.34) and pay and promotion equity (*r* = −30). Patient care attitudes was also positively related to patient care practices (*r* = 0.47) and negatively related to CWB (*r* = 1.00). Results for CWB should be interpreted with caution due to small sample size.

As shown in Table 3, microaggressions was positively related to imposter phenomenon (*b* = 0.24, *t* (116) = 2.41, *p* = 0.02) but not gender identity salience ((*b* = 0.004, *t* (116) = 0.65, *p* = 0.52), therefore H1 was partially supported. For H2, the negative relationship between self-reported patient care practices (*b* = −0.01, *t* (94) = 1.49, *p* = 0.14) and patient care attitudes (*b* = 0.00, *t* (93) = 0.30, *p* = 0.77) was not supported. Consistent with H3, the positive relationship between microaggressions and CWB (*b* = 0.19, *t* (23) = 4.38, *p* = 0.00) was supported, however, results should be interpreted with caution due to the low sample size. Lastly, the negative relationship between microaggressions and perception of promotion and pay equity (*b* = −0.03, *t* (116) = −5.05, *p* = 0.00) (H4) was supported.

**Table 3.** Total Effects of Microaggressions on Employee Outcomes While Controlling for Race, Age, and Training Level

	Imposter syndrome		Gender salience		Patient safety		Patient care		Counterproductive work behavior		Perception of promotion and pay equity	
	*b *(SE)	*Δ*R^2^	*b *(SE)	*Δ*R^2^	*b *(SE)	*Δ*R^2^	*b *(SE)	*Δ*R^2^	*b *(SE)	*Δ*R^2^	*b *(SE)	*Δ*R^2^
Step 1 Race	1.27 (0.86)	0.21^***^	0.04 (0.05)	0.01	−0.06 (0.09)	0.03	−0.01 (0.07)	0.06	0.55 (0.35)	0.10	−0.04 (0.05)	0.09^*^
Age	−0.6.5 (1.74)		−0.09 (0.10)		−0.16 (0.19)		−0.23 (0.14)		−0.53 (0.65)		−0.31 (0.10)	
Training level	−1.79 (2.28)		0.15 (0.13)		−0.16 (0.24)		−0.02 (0.18)		−0.40 (0.92)		−0.05 (0.14)	
Step 2 Microaggression	0.24 (0.10)	0.04^*^	0.00 (0.01)	0.00	−0.01 (0.01)	0.02	0.00 (0.01)	0.00	0.19 (0.04)	0.46^***^	−0.03 (0.01) ^***^	0.00^***^

^*^ *p* < 0.05; ^**^p < 0.01; ^***^p < 0.001.

*b*, unstandardized regression coefficient; SE, standard error.

## Discussion

### Microaggressions and imposter phenomenon/CWB

Unsurprisingly, many female physicians report having experienced microaggressions in the workplace—84.6%. In the past 6 months, 38.7% of respondents felt that they could not contribute to the conversation in the workplace and 35% felt unheard in the past 6 months. Microaggressions were associated with an increased risk of both imposter phenomenon and CWB. This has been reported previously in the literature in a study of gastroenterologists. Sears et al. found that imposter phenomenon can evolve into CWB, which is exhibited by fears of asking for help, avoiding leadership positions or the need to strive for perfectionism.^23^

### Microaggressions and promotion/pay equity

Microaggressions have also had a negative association with pay and promotions equity. Female gender has been linked to a lower chance of promotion and pay equity. In a 15-year longitudinal study of several academic institutions, women when compared with men had a lower chance of achieving a senior leadership position despite high productivity.^24^ This discrepancy is even worse for faculty of Color, especially those who are Black or Asian.^25^

To be promoted, one needs to have many publications. Women are less likely to be invited to write articles and are also less likely to be last author indicating senior authorship.^26^ Women are also less likely to serve on editorial boards for journals. In a study of six major surgical journals, those who served on the editorial board are more likely male (74%) and white (75%).^27^ In another study of 42 surgical journals, only 14.8% (*n* = 420) of women were found to serve on editorial boards and only 4.8% (*n* = 2) served as editor-in-chief.^28^ External speaking engagements are also needed for promotion; unfortunately, female physicians are also less likely to give grand rounds as compared with their male counterparts.^29^

Even when given these opportunities, women are more likely to be introduced by their first name as opposed to male presenters who are more often introduced as doctor.^30^ This practice is a microinvalidation as it reinforces the stereotype that women are less than their male counterparts, which can further add to a female physician's imposter syndrome.

### Limitations

There were several limitations to this study, including the cross-sectional study design. A cross-sectional study design indicates that there is a relationship between microaggressions and imposter phenomenon, CWB, perception of pay, and promotion equity; however, it does not indicate that microaggressions *cause* female physicians to react negatively which, in turn, may affect their attitudes toward patient care practices and attitudes. Future studies should explore the relationship between these variables and microaggressions using other research methodology, such as longitudinal studies and experimental designs to determine causality. Longitudinal designs would allow for the assessment of exposure to microaggressions over time.

Also, there may have been selection bias among those who agreed to participate, as the first author is an internal medicine subspecialty attending and a URM, which may have contributed to the overwhelmingly higher responses from internal medicine specialists and subspecialists, as well as the response rate of those who identify URM. A majority of respondents were attendings as opposed to residents or fellows; perhaps there was fear of retribution among trainees.

The small sample size also limited the analysis, which limits its generalizability. Additionally, nearly 50% of respondents were white, which limited the analysis examining the association between race and microaggressions as well as secondary variables. In academic medicine, microaggressions are experienced more by females than males, especially those of non-white race/ethnicity.^31,32^

Using only female respondents prevented us from exploring microaggressions that male physicians may encounter. A previously conducted study examining microaggressions in academic medicine used both male and female participants but 80% of them were white, which limited the findings.^31^ Men who belong to marginalized groups such as ethnic minority or Lesbian, Gay, Bisexual, Transgender, Queer, Intersex, Asexual may have different responses, thus limiting the generalizability of the responses in that study or this study.

## Conclusions

In this cross-sectional analysis of female medical and surgical physicians in our health system, nearly 90% of respondents report having experienced microaggressions. Those who report experiencing microaggressions were more likely to report having imposter phenomenon and performing CWB, as well as a lack of perceived promotion and pay equity. As a result, women in academia need to be supported to overcome the burdens of imposter phenomenon, CWB, and pay/promotion equity. There also needs to be consideration of intersectionality as URM females face both gender and racial discrimination. Means of support for female physicians include mentoring, sponsorship, and/or career development. Institutions also need to implement policies to create a more inclusive workplace for female physicians.

## Supplementary Material

Supplemental data

## Supplementary Material

Supplementary Appendix SA

## Abbreviations Used

CIPS: Clance Imposter Phenomenon Scale
CWB: counterproductive work behavior
CWB-C: Counterproductive Work Behavior Checklist
*M*: mean
OB-GYN: obstetrics and gynecology
PM & R: physical medicine and rehabilitation
SD: standard deviation
SE: standard error
URM: underrepresented minorities
