# Supplementary material for: Intersectionality and Its Impact on Microaggression in Female Physicians in Academic Medicine: A Cross-Sectional Study
Source: Womens Health Rep (New Rochelle). 2023 Jun 26;4(1):298–304. doi: 10.1089/whr.2022.0101 (PMC10316039; doi:10.1089/whr.2022.0101)
Supplement: Supplemental data [file Suppl_AppendixSA.docx]

**Supplemental Data: Survey given to all participants**

**Demographics**

**Age**

25-35

36-45

46-55

Over 55

**Race**

White

Black

Asian

Mixed

Other

**Ethnicity**

Hispanic

Non-Hispanic

**Level of Training**

Resident

Fellow Attending

Attending

**How many years ago did you graduate from medical school?**

5 years or less

6-10 years

11-15 years

15 years or more

**What is your main area of practice?**

Internal Medicine

Internal Medicine Subspecialty

Surgery

Surgery Subspecialty

Psychiatry

Anesthesia

Radiology

Radiation Oncology

Pediatrics

Dermatology

Obstetrics and Gynecology

Urology

Ophthalmology

*Micro-aggression is defined as subtle snubs, slights, and insults directed towards minorities, as well as to women and other historically stigmatized groups, that implicitly communicate or at least engender hostility.” This definition extends beyond verbal abuse to include general disrespect, devaluation, and the exclusion of recipients. Microaggressions occur daily and are commonly delivered automatically with dismissive body language and tone of voice.* **The Merriam-Webster dictionary defines a microaggression as: “a comment or action that subtly and often unconsciously or unintentionally expresses a prejudiced attitude toward a member of a marginalized group (such as a racial minority). In your current position, have you ever been the victim of a microaggression?**

Yes

No

**If yes, can you give an example: ________________________________________________________ ______________________________________________________________________________________________________________________________________________________________________**

**Gender bias scale:**

Likert scale ranging from 1 = strongly disagree to 5 = strongly agree. Frequency scale (indicated by *) ranges from 1 = never to 5 = always

1. I have been asked to do a job that everyone knew was likely to fail.
2. I have been held responsible for organizational problems outside of my control
3. Women in my organization seem to be given leadership roles with a high risk of failure
4. In my organization, there is pressure to conform to gender stereotypes
5. People in my organization assume that top leadership will be men
6. The decisions in my organization are made by men
7. The “boys club” mentality is present in my workplace
8. Even though my spouse/partner does not work for my organization, s/he is expected to host invents
9. My organization expects spouses/partners of senior leaders to contribute to unpaid volunteers
10. My organization vets spouses/partners of senior leaders as part of the hiring process
11. I am mindful of my communication approach when exercising authority at work
12. I wait to be acknowledged prior to speaking in a meeting
13. I am caution when self-promoting at work
14. I downplay my accomplishments when speaking to others
15. I chose my field of study because it was considered suitable for women
16. I would have chosen a different field, but it was considered inappropriate for women
17. Growing up I was encouraged to pursue certain careers that were appropriate for women
18. My ideas seem more likely to be taken seriously when a man repeats them
19. My jo performance has been scrutinized more closely than that of my male colleagues
20. As a woman I am expected to be nurturing at work
21. I work harder than my male colleagues for the same credibility
22. I feel welcomed while attending social events with my male colleagues
23. Male colleagues socialize without me*
24. I have been excluded from leadership events (e.g., off-sites, retreats) because of my gender*
25. I have received significant mentoring
26. I have had a female mentor
27. I have had to learn how to lead on my own
28. Other leaders have recommended me for advancement opportunities
29. I have had another leader sponsor me for promotion
30. At work, I am interrupted by men when I am speaking*
31. When I am the only women in a meeting, I find it difficult to gain support for my ideas*
32. It is taken for granted when I help my male colleagues with their responsibilities*
33. My efforts at creating harmony at work are noticed
34. I have made less money than my male counterparts
35. I have made less money than men who have held my position prior to me
36. I have had opportunities blocked by other women at work
37. Women in higher positions have made my job more difficult
38. High-level women in my organization protect their turf
39. High-level women in my organization help other women succeed
40. I have experience verbal abuse at work
41. The behavior of my male co-workers has sometimes made me feel uncomfortable
42. I have been sexually harassed at work
43. I speak up about challenges women face at work*
44. I advocate for women’s rights at work*
45. It requires the encouragement of others for me to accept a new opportunity*
46. I have turned down a promotion because I have felt unqualified
47. My personal obligations have prevented me from pursuing opportunities for advancement.

**Gender Micro-aggressions**

Responses: items range from 0 (*not at all stressful) to* 5 (extremely stressful). Frequency items ranged from 0 (never) to 5 (once a week or more). 1 = I did not experience this event to 5 = I experience this event 7 or more times.

1. Someone made me feel unattractive because of the size of my butt
2. Negative comments about the size of my facial features
3. Imitated the way they think a Black woman (women of my race) speak
4. Someone made me feel unattractive
5. Negative comment about skin tone
6. Someone assumed I speak a certain way
7. Objectified me based on physical features
8. Someone assumed I have a certain body time (stress only)
9. Made a sexually inappropriate comment
10. Negative comments about my hair when natural
11. Assumed I was sexually promiscuous (frequency only)
12. I have felt unheard
13. My comments have been ignored
14. I have been disrespected in the workplace
15. Someone had tried to put me in my place
16. Felt excluded from networking opportunities
17. Assumed I did not have much to contribute to the conversation
18. Someone assumed I was sassy and straightforward (stress only)
19. I have been told that I am too independent
20. Someone made me feel exotic as a Black woman (stress only)
21. I have been told I am too assertive
22. Assumed to be a strong black woman
23. Someone has told me to calm down
24. Perceived to be “angry black woman”
25. Someone accused me of being angry when speaking calm

**Clance Imposter Syndrome Scale**

*Directions: For each question, please circle the number that best indicates how true the statement is for you. It is best to give the response that enters your mind rather than dwelling on each statement and thinking about it over and over.*

Scoring: 1 = not at all true; 2 = rarely; 3 = sometimes; 4 = often; 5 =very true

1. I have often succeeded on a test or task even though I was afraid I would not do well before I undertook the task
2. I can give the impression that I’m more competent than I really am.
3. I avoid evaluations if possible and have dreaded others evaluating me
4. When people praise me for something I have accomplished, I’m afraid I won’t be able to live up to their expectations of me in the future
5. I sometimes think I obtained my present position or gained my present success because I happened to be in the right place at the rite time or knew the right people
6. I am afraid people important to me may find out that I am not as capable as they think I am
7. I tend to remember the incidents in which I have not done my best than those time I have done my best
8. I rarely do a project or task as well as I’d like to do it.
9. Sometimes I feel or believe that my success in life or in my job is the result of some kind of error.
10. It’s hard for me to accept compliments or praise about my intelligence or accomplishments
11. At times, I feel my success has been due to some kind of luck
12. I’m disappoint at times in my present accomplishments and think I should have accomplished much more
13. Sometimes I’m afraid other will discover how much knowledge or ability I really lack
14. I’m often afraid that I may fail at a new assignment or undertaking even though I generally do well at what I attempt.
15. When I’ve succeeded at something and received recognition for my accomplishments, I have doubts that I can keep repeating that success.
16. If I receive a great deal of praise and recognition for something’ I’ve accomplished, I tend to discount the importance of what I’ve done.
17. I often compare my ability to those around me and think they may be more intelligent than I am.
18. I often worry about not succeeding with a project or examination, even though others around me have considerable confidence I will do well.
19. If I’m going to receive a promotion or gain recognition of some kind, I hesitate to tell others until it is an accomplished fact
20. I feel bad and discouraged if I’m not “the best” or at least “Very special in situations that involve achievement

Which is more important to you: race, gender, religion, sexual orientation, ethnicity, culture/country of origin.

**Identity Salience Measure**

*Social identity salience refers to the extent to which a particular social identity (e.g., race/ethnicity, gender, sexual orientation, religion, culture/country of origin) represent an integral part of who you are relative to other social roles.*

1. *Please select your most salient social identity*
   1. *Race/ethnicity*
   2. *Sexual orientation*
   3. *Gender*
   4. *Religion*
   5. *Culture/country of origin*

*Please rate the extent to which you agree, Likert scale ranging from: 1 = strongly disagree to 7 = strongly agree*

1. Overall, being a woman has very little to do with how I feel about myself
2. In general, being a woman is an important part of my self-image
3. My destiny is tied to the destiny of other women
4. Being a woman is unimportant to my sense of what kind of person I am
5. I have a strong sense of belonging to other women
6. I have a strong attachment to other women
7. Being a woman is an important reflection of who I am
8. Being a woman is not a major factor in my social relation.

**Outcomes**

**Self-reported suboptimal patient care practiced at least month**

*Directions: rate how frequently you found yourself exhibiting the following attitudes or behaviors for any reason.*

*Response: 1 = never, 2 = once, 3 =several times per year, monthly, weekly*

1. I found myself discharging patients to make the service “manageable” because the team was busy
2. I did not fully discuss treatment option or answer a patient’s questions
3. I made treatment or medication for an agitated patient without evaluating him or her
4. I did not perform a diagnostic test because of desire to discharge a patient

self-reported suboptimal patient care attitudes experienced in a month

1. I paid little attention to the social or personal impact of an illness on a patient
2. I had little emotional reaction to the death of one of my patients
3. I felt guilty about how I treated a patient from a humanitarian standpoint.

**Counter-productive Work behavior**

| **How often have you done each of the following things on your present job?** | Never  Once or twice  Once or twice/month  Once or twice/week  Every day |
| --- | --- |
| 1. Purposely wasted your employer’s materials/supplies | 1 2 3 4 5 |
| 2. Complained about insignificant things at work | 1 2 3 4 5 |
| 3. Told people outside the job what a lousy place you work for | 1 2 3 4 5 |
| 4. Came to work late without permission | 1 2 3 4 5 |
| 5. Stayed home from work and said you were sick when you weren’t | 1 2 3 4 5 |
| 6. Insulted someone about their job performance | 1 2 3 4 5 |
| 7. Made fun of someone’s personal life | 1 2 3 4 5 |
| 8. Ignored someone at work | 1 2 3 4 5 |
| 9. Started an argument with someone at work | 1 2 3 4 5 |
| 10. Insulted or made fun of someone at work | 1 2 3 4 5 |

**Perception of pay and promotion equity**

*Please select the extent to which you agree or disagree with the following statements*

*Rating scale: 1 = strongly disagree to 5 = strongly agree*

1. My salary and opportunity for promotion is fair given my qualifications for my position.
2. My salary and opportunity for promotion is fair in relation to all other physicians in the health system
3. My salary and opportunity for promotion is fair in relation to other physicians with comparable qualifications at other institutions.
